# MUP: Simplifying Secure Over-The-Air Update with MQTT for Constrained IoT Devices

**DOI:** 10.3390/s21010010

**Published:** 2020-12-22

**Authors:** Kristina Sahlmann, Vera Clemens, Michael Nowak, Bettina Schnor

**Affiliations:** Institute of Computer Science, University of Potsdam, August-Bebel-Str. 89, 14482 Potsdam, Germany; vera.clemens@st.ovgu.de (V.C.); minowak@uni-potsdam.de (M.N.)

**Keywords:** Internet of Things, security, firmware update, MQTT, edge computing

## Abstract

Message Queuing Telemetry Transport (MQTT) is one of the dominating protocols for edge- and cloud-based Internet of Things (IoT) solutions. When a security vulnerability of an IoT device is known, it has to be fixed as soon as possible. This requires a firmware update procedure. In this paper, we propose a secure update protocol for MQTT-connected devices which ensures the freshness of the firmware, authenticates the new firmware and considers constrained devices. We show that the update protocol is easy to integrate in an MQTT-based IoT network using a semantic approach. The feasibility of our approach is demonstrated by a detailed performance analysis of our prototype implementation on a IoT device with 32 kB RAM. Thereby, we identify design issues in MQTT 5 which can help to improve the support of constrained devices.

## 1. Introduction

Internet of Things (IoT) devices (further referred to as devices) are cyber-physical objects which are equipped with sensors and actuators and are connected to the Internet. They can be constrained in terms of computational power, memory, network bandwidth and energy. Billions [1] of such devices are used meanwhile in home automation and industrial domains such as agriculture and manufacturing. In case a security exploit is identified, the firmware should be fixed as soon as possible. Otherwise, the risk exists that these IoT devices are hijacked and misused, e.g., as a botnet. Hence, a firmware update solution for such devices is essential to deal with vulnerabilities [2].

Further, it must be ensured that patches and updates are only obtained from trustworthy sources. In the update process, there are two main security properties to prove:

1. *Authenticated firmware:* The device has to be able to verify that the received firmware is sent by a trustworthy source.
2. *Freshness of the firmware:* The freshness property claims that the device has to be able to verify that the new firmware has a higher version number than the installed firmware.

The freshness property is even more important in the IoT: To save energy, only *fresh* updates should be transferred to the IoT device. Hence, the freshness of the firmware should be proven before the firmware transmission is started. The freshness property also prevents replay attacks.

We propose and evaluate an update protocol for IoT devices managed by MYNO [3] which is based on the open protocols Message Queuing Telemetry Transport (MQTT) [4] and the Network Configuration Protocol (NETCONF) [5]. MQTT is a common protocol in the IoT domain [6]. The NETCONF protocol is an Internet Engineering Task Force (IETF). specification for the configuration management of network devices. In the MYNO architecture, the IoT devices are separated from the Internet via a gateway running on the edge node. Since the edge node is a more powerful device compared to the IoT devices, it is suited to overtake more complex and energy-consuming tasks, for example to distribute firmware updates.

The main scientific contributions of this paper are:

- We propose an MQTT-based architecture for IoT management and show how the update process is integrated.
- The proposed MYNO Update Protocol (MUP) is suited for constrained devices which is demonstrated with our prototype on an IoT device with only 32 kB RAM and the firmware transmission over 6LoWPAN.
- We assess the security properties of MUP in Section 4 and show that MUP fulfills the security properties *authenticated* and *fresh firmware* and is also safe against replay attacks.
- The implementation challenges are discussed in Section 5. Especially, the need of slicing is motivated.
- We give a detailed analysis of the communication overhead regarding MQTT and 6LoWPAN (see Section 6).
- We discuss the implementation issues in Section 7 and identify optimization potential in MQTT implementations and the MQTT standard to further improve the support of constrained devices.

Before we present and evaluate the MYNO Update Protocol (MUP) in Section 3, the next section discusses the security properties of related update protocols from the literature and their suitability for constrained devices.

## 2. Related Work

Samuel et al. [7] present The Update Framework (TUF) which builds the basis of the update system Thandy [8]. Thandy was originally developed for secure updates for the Tor project [9]. The design of TUF focuses on the security principle *survivability*, defined as the ability of the system to function correctly while under attack or partial compromise. For better resilience against key compromise, they propose the separation of duties and multi-signature trust. Multi-signature trust may be achieved by signatures of multiple roles or by threshold signatures, where at least *t* signers are required out of a set of *n* potential signers. Further, TUF uses a two-step approach where signed metadata describing the new update is downloaded and checked first before the update file is downloaded and installed. This two-step approach is also very suited for the update of constrained devices and used in the presented MUP protocol (see Section 3).

Uptane [10], a software update system for automobiles, adapts TUF in order to address the specific automotive requirements. For example, it adds a director role at the repository site to blacklist faulty software and for customizing software when the vehicle owners may have paid for extra features. Further, they combine TUF with an edge computing architecture by adding the concept of primary and secondary components. The primary is connected to the software repository, downloads the new update and distributes it to the secondary components.

Then there are several research groups investigating update protocols for IoT devices. Some of them propose MQTT-based solutions ([11,12,13]) and others propose customized/proprietary solutions [14,15]. Further, we discuss work in progress at the IETF [16,17,18].

The Open Mobile Alliance has specified a TLS-based update process within the Lightweight Machine-to-Machine (LwM2M) protocol [19]. LwM2M is based on the Constrained Application Protocol (CoAP) [20] and uses a pull approach where the device/client periodically polls a server for new updates. Then the client connects to the URI provided by the LwM2M server and downloads the firmware. In this architecture the devices need Internet access, while in the proposed MYNO architecture the constrained devices are separated from the Internet by the edge node.

Thantharate et al. [11] compare CoAP and MQTT for delivering software and security updates. They argue that in the IoT the constrained devices are meant to last for a number of years with limited power, and therefore most of the time the devices will be inactive (sleeping). For transmitting a new firmware, a robust transport of the data is necessary. Therefore, Thantharate et al. evaluate the performance of MQTT and CoAP for reliable data transport. They compare MQTT with Quality of Service (QoS) level 1 (deliver the message at least once, with confirmation required) and QoS level 2 (exactly-once) against CoAP in CON mode (Confirmable messages). They use a simulator in their performance study. The results show that MQTT performs faster and has fewer spikes in transmission durations due to retransmissions. This is not surprising, since MQTT uses TCP, while CoAP uses UDP. Hence, they recommend MQTT for IoT updates. While Thantharate et al. draw their conclusion from simulation results, this work presents results with a real implementation of an update framework.

Langiu et al. [15] have recently proposed a new update protocol called UpKit dedicated for IoT devices. UpKit installs authenticated firmware and guarantees the freshness of the update. The benefit of that framework is that freshness is guaranteed without the use of an Network Time Protocol (NTP) server and authenticated clocks. The evaluation shows that UpKit has a small memory footprint. Further optimizations are the support for A/B updates and the support of differential updates. A/B updates require two bootable slots which keep two images, and the bootloader jumps to the newest slot. Differential updates reduce the amount of data which have to be transferred over the network which saves energy and the actual update time. The UpKit implementation supports CoAP or Bluetooth Low Energy [21] for communication. Our proposed MUP adapts this approach for an MQTT-based IoT environment.

Frisch et al. [13] also consider an over-the-air (OTA) update process via MQTT, but present no performance numbers. They experiment with the ESP8266 micro-controller board which has 96 kB main memory and integrated WiFi. Instead, we investigate the OTA update procedure for much more constrained devices and networks. Further, their approach does not guarantee the freshness of the firmware (version numbers are sent in clear text and not signed). Another weakness is that the firmware verification is done after download, but that is too late when dealing with constrained devices. If the verification fails, the device has spent much energy for the transmission of the malicious firmware. This makes Denial of Service (DoS) attacks possible. MUP avoids this weakness by a two-phase approach similar to UpKit [15] and TUF [7].

An approach for the secure distribution of firmware using MQTT is proposed by Lo and Hsu [12]. However, the MQTT protocol is only used between the firmware patch server, the firmware broker server and the gateway. The gateway is connected to the Internet and communicates with the IoT devices via wireless connections such as Wi-Fi or Bluetooth. The protocol between gateway and device is not further specified. In opposite, the proposed MUP protocol relies on MQTT for the communication with the devices. Further, while MUP only needs to pre-install the public vendor key, in the approach of Lo and Hsu one secret value and one secret key have to be pre-installed on the devices.

Laukkarinen et al. [14] present the design and implementation of a firmware update protocol for resource constrained Wireless Sensor Nodes (WSN). They propose the use of a Message Authentication Code (MAC) for integrity checking. This needs shared secret keys between update server and device. Again, this approach is not scalable for vendors. Instead, MYNO uses signatures, and therefore only public keys have to be distributed.

There is work in progress at the IETF [16,17]. The draft for software updates for IoT (SUIT) [16] assumes asymmetric cryptography and a public key infrastructure. A data structure called manifest [17] specifies 24 elements with detailed information about the firmware. The manifest has an optional Expiration Time, but this needs a secure source of time which is not available on most IoT devices. Instead, our proposed MYNO Update Protocol uses a Nonce to avoid replay attacks. Zandberg et al. [18] implemented and evaluated a prototype to compare the surveyed firmware update methods, among them the SUIT-OTA update. They use CoAP blockwise transfer to pull the firmware image onto the device.

The following section presents the MYNO Update Protocol which demonstrates how the concept of UpKit can be transferred to an MQTT-based IoT environment.

## 3. Update Over-The-Air (OTA) with MYNO

**Figure 1:**
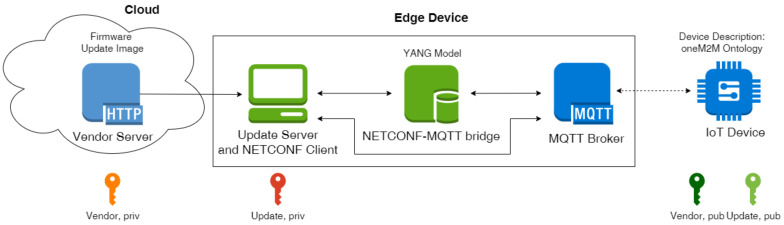
System architecture of the MYNO framework.

In [3], Sahlmann et al. introduced ontology-driven device descriptions. The corresponding architecture, in the following called MYNO, consists of an MQTT [4] broker, the YANG [22] model for data modeling, and the NETCONF [5] protocol for device management as shown in Figure 1.

The MQTT protocol follows the publish/subscribe paradigm and is used for the communication between IoT devices and the edge of the network where the NETCONF client runs. The web-based NETCONF client acts as a user interface. The benefit of using NETCONF is that this protocol is an open standard and any NETCONF client can configure devices into a network. On the other side however, it has been shown that the NETCONF server cannot be installed on a constrained IoT device due to its limited resources [23,24]. Therefore, MYNO introduces the NETCONF–MQTT bridge which translates between the two protocols, namely between Remote Procedure Calls (RPCs) of NETCONF and MQTT messages.

The NETCONF protocol provides only operations for network configuration. However, further operations can be added by RPCs. Such device capabilities are described in the semantic device description [3]. The MYNO device descriptions are based on the oneM2M Base Ontology [25], represented in the Web Ontology Language (OWL) [26]. OWL does not only provide a standard for structuring vocabulary, but also has some main advantages:

- information is represented in a formal, machine-readable way;
- the W3C SPARQL [27] query language (i.e., SQL-similar notation) can be used for search on certain individuals using classes and relationships (e.g., used for parsing device descriptions in the NETCONF–MQTT bridge).
- a reasoner can check consistency and infer new information (e.g. aggregation of services in a Virtual Device [28]);
- two concepts in different ontologies with similar meaning can be mapped (i.e., using the property owl:sameAs), for example if another ontology than the oneM2M base ontology is used in the device description.

Therefore, data described by an ontology help to understand the domain of interest, can be processed in a structured way, and new facts can be inferred. Hence, the device capabilities can be described by an ontology defined for a certain domain.

During the *bootstrap process*, the device description is published by an IoT device to the MQTT broker. The NETCONF–MQTT bridge parses this ontology and generates the YANG data model with corresponding RPC operations. The device description has to be extended for the update capabilities. After the bootstrap process, the update functionality is activated in the NETCONF client.

This paper shows how a secure update process can be integrated into the MYNO architecture.

### 3.1. Prerequisites

Private and public keys must be distributed before the update process starts. The vendor possesses a Private/Public key pair $\left(K_{pub}^{Vendor},K_{priv}^{Vendor}\right)$, and pre-installs the vendor public key $K_{pub}^{Vendor}$ on the device. The Update Server possesses also a Private/Public key pair $\left(K_{pub}^{Update},K_{priv}^{Update}\right)$ and propagates its public key $K_{pub}^{Update}$ to the device during the bootstrap process (see key distribution in Section 4).

### 3.2. MYNO Update Protocol (MUP)

We adapt the UpKit approach (see Section 2) where the Update Server at the edge verifies the freshness of the firmware before it is transmitted to the device.

We designed the update process as an push approach. The network administrator initiates the download of a new firmware image from the vendor server (see Figure 1). The vendor provides the firmware, and a so-called vendor manifest (see Table 1) which describes the firmware image characteristics such as size and version number. The manifest includes also a vendor signature called *inner signature*.

**Figure 2:**
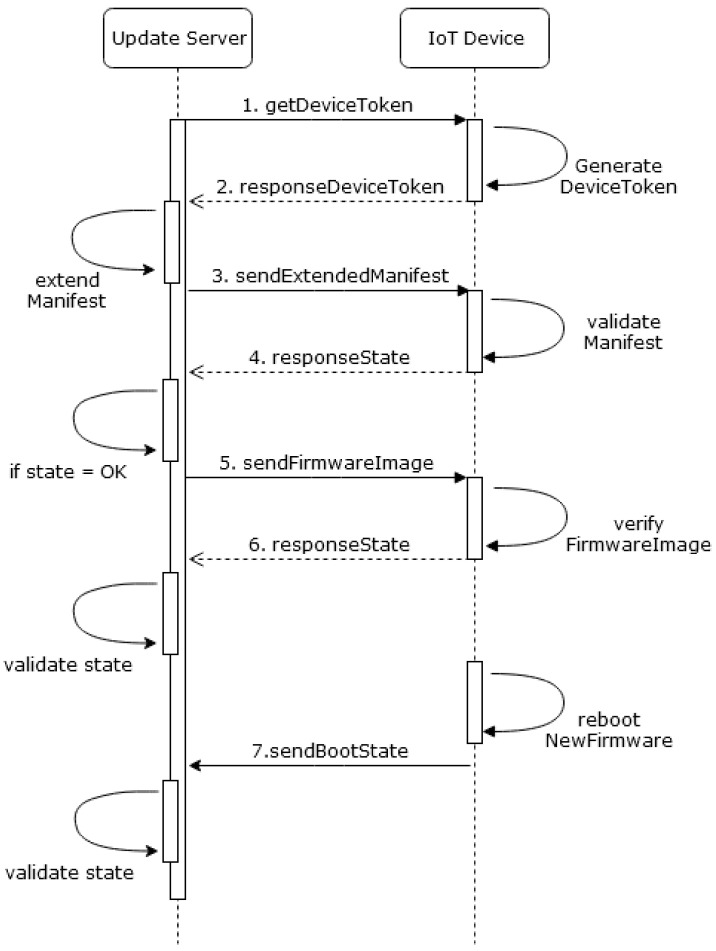
MYNO update protocol as a sequence diagram.

The MYNO update protocol is shown in Figure 2. For simplicity, we omit the components NETCONF–MQTT bridge and the MQTT broker because they are agnostic to the messages and act only as intermediaries during the update process. Messages starting with response are sent as a MQTT response which were introduced in MQTT v5 [4]. The update protocol works as follows:

(1) The Update Server requests a device token from the IoT device.
(2) The device token contains the device Universally Unique Identifier (UUID), the current version of the firmware, and a nonce (see Table 2). The generated token is sent within a response.
(3) The device token information is used to generate the extended manifest which grants the freshness of the update: The Update Server appends the device UUID, version and the nonce from the device token to the vendor manifest and signs this extended manifest with his private key (see Table 1). Now the extended manifest carries a double signature. The extended manifest is then sent to the device for validation.
(4) The device validates the extended manifest using the public keys of the vendor and Update Server. The following fields are checked for the freshness of the firmware: nonce and device UUID must be the same as sent before with the device token. Further, the new version must be higher. The old version is required for differential updates only. If the extended manifest was successfully validated, the device responds with the state ok.
(5) If the responseState was ok, the Update Server starts the transmission of the firmware image.
(6) When the firmware is fully transmitted, the device performs an integrity check: It calculates a digest of the firmware and compares it with the digest included in the manifest. Since the digest in the vendor manifest was correctly signed by the vendor, this proves the authenticity of the firmware. If this verification is successful, the device responds with the state ok.
(7) The device reboots with the new firmware and notifies the Update Server about success.

If the validation of the manifest or the firmware is not successful, the update process will be cancelled by the device. If the reboot fails because of other reasons, the Update Server will get a timeout and reports the error.

## 4. Security Discussion

In this section, we show that MUP achieves the security properties defined in Section 1. Further, we discuss its robustness against resource exhaustion. However, the security aspect is linked with configuration and management effort. Therefore, we also discuss MUP’s key distribution process.

### 4.1. Guaranteed Security Properties

The proposed MYNO update protocol achieves both security properties: an authenticated firmware and freshness of the firmware.

Two steps are necessary for the authentication of the new firmware. First, the device validates the vendor manifest using the public key of the vendor. If the validation is correct, the device has trust into the digest of the manifest (which is the hash value of the firmware update). In a second step after the download of the firmware, the device checks whether the received firmware corresponds to the manifest. Therefore, it calculates the firmware digest and compares it with the digest in the manifest. If they are the same, the device has also trust in the firmware.

The new version number is signed by the vendor in the vendor manifest and checked by the device whether it is higher than the current version number. Further, the freshness of the firmware is guaranteed by the double signature process where a nonce is generated by the device and signed by the Update Server in the extended manifest. Since also the device UUID is included in the signature, this challenge is unique for each device.

### 4.2. Replay Attacks

The MUP protocol does not rely on TLS. All messages are sent in clear text. Hence, an adversary may resend these messages to initiate more firmware updates. Even installing the same firmware again and again would be a DoS attack ending when the device battery is empty. Lo and Hsu [12] rely only on signed version numbers to guarantee the freshness of the firmware. MYNO follows the UpKit approach and use nonces to verify the freshness of the firmware. This hardens the protocol against First-Pre-Image-attacks since the time for an attacker to prepare such an attack is shortened. Further, the window for the DoS attacks is minimized in MUP because a device subscribes to the topic for the firmware image just before receiving it (before step 5) and unsubscribes as soon as the firmware image is received (after step 6).

### 4.3. Confidentiality

There may be several reasons why a firmware vendor may prefer to send the firmware update encrypted. First, this may be important due to licensing. Since the transmitted firmware is opaque to MUP, the vendor may send the firmware update encrypted and the Update Server forwards it to the device. However, this assumes appropriate keying material on the device.

Alternatively, the connection channel can be encrypted. The channel between vendor and update server may be secured by TLS [29]. The MUP messages may also be sent encrypted by MQTT over TLS, but this has to be supported by the device. For example, the Arduino Nano 33 IoT supports MQTT over TLS, while Contiki-NG does not support it [30].

Since support for efficient encryption is considered as an important feature, crypto chips are getting more wide-spread in IoT devices. For example, the Arduino Nano 33 IoT [31] is equipped with the crypto chip ATECC608A. The crypto chip has a data zone where up to 16 keys or compressed certificates may be stored [32].

### 4.4. Edge Architecture and Man-in-the-Middle Attacks

In cloud based solutions like the Arduino IoT Cloud [33], AWS IoT [34] or IBM IoT Cloud [35], the devices are directly connected to the Internet. In contrast, MYNO relies on an edge computing architecture where the IoT devices are separated from the Internet via a gateway. Hence, all Internet connections are terminated at the edge node.

If an attacker breaks into the edge node system, he has a powerful man-in-the-middle position and may for example inhibit software updates. Hence, the edge node has to be managed with the same care as every other machine which has Internet connection.

### 4.5. Key Distribution and Update

The MUP protocol requires two public keys on the devices: the pre-installed public vendor key and the public key of the Update Server. While mechanisms for key distribution and key update are important building blocks belonging to a security architecture, we do not focus on this topic here. A survey of key bootstrapping protocols in the Internet of Things based on public-key cryptography can be found in [36].

Lo and Hsu [12] propose Diffie–Hellman for key exchange, since they have to create different keys for every device. Instead of managing secrets for every device, MUP uses public/private key pairs. Only the public key of the vendor has to be pre-installed, and the public key of the Update Server is propagated during the bootstrap phase. This makes MUP scalable.

**Figure 3:**
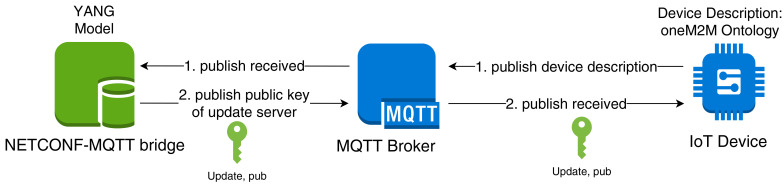
Bootstrap process.

Currently, MYNO uses the bootstrap process also for the distribution of the key of the Update Server. The bootstrap process consists of two steps (see Figure 3): (1) when a new device enters a network, it publishes its device description; the NETCONF–MQTT bridge parses this description and adds the device to the YANG model; (2) if successful, the bridge publishes this state and the public key of the Update Server to the response topic of the device. Obviously, this approach is vulnerable against eavesdropping. In case an attacker is able to reply faster than the update agent to the first message, the device will take over the wrong key and get compromised, too.

In the Arduino IoT Cloud solution [33] the Arduino certificate is stored as trust anchor on the device. During bootstrap the Arduino client sends a *Certificate Signing Request* to the Arduino Cloud to generate a client certificate. In a similar way, the bootstrap phase of MYNO can be improved by using self-signed certificates where the vendor certificate is used as a trust anchor. The Update Server has to be equipped with a certificate signed by the vendor. This certificate can be verified by the device using the pre-installed vendor certificate.

An update of the vendor key may be supported by the Update Server running on the edge. For the verification of the new vendor key, the Update Server may employ DNSSec/DANE [37,38].

### 4.6. Robust against Resource Exhaustion

Considering the constraints of IoT devices in terms of network bandwidth, memory, storage and energy, the update process must ensure that no unnecessary data transmissions and reboot occurs. The proposed MUP protocol ensures this by two steps: In the first phase, only the extended manifest is transferred to the device and checked. The extended manifest is much smaller than the new firmware. If the validation of the manifest guarantees freshness, the firmware will be downloaded in the second phase. This separation avoids unnecessary transfers and reboots.

Since an attacker could periodically send manifests promising a new update without a valid signature, the signature verification process may drain the battery. Since MUP is built upon the UpKit approach and uses an extended manifest, the device checks first the manifest extension carrying the Nonce. A correct signature of the manifest extension authenticates the Update Server as the origin.

### 4.7. Usability

The additional effort on the vendor side is minimal: The devices have to be pre-installed with the following components: the device description, the public vendor key, the application and bootloader. Further, the bootstrap protocol has to support the exchange of the public key of the Update Server.

The presented vendor manifest and the manifest extension carry no information about the used crypto algorithms. This approach is not feasible in a productive environment with devices from different vendors. Hence, the vendor manifest has to be extended with this information. At the IETF, there are efforts underway for standardizing a suited manifest that describes the firmware image and processing steps [17]. This approach may also be combined with MUP.

MUP has been easily integrated into the existing MYNO architecture. Only a few adjustments were necessary on the bridge (e.g., adding a binary data type for signatures for new parameters). The update server is an extension of the NETCONF client for the web-interface.

The automated distribution of update images to IoT devices is relevant to prevent security gaps. MUP can be easily integrated into the DevOps processes and Continuous Delivery (CD) pipeline using scripts.

## 5. Implementation

In normal operation mode, an IoT device is sending data to the edge. In case of a firmware update, several kilobytes of data have to be transferred to the device. This is not a common task in the IoT and brings challenges for the communication layer.

This section describes the details of the MUP implementation in the MQTT-based MYNO architecture. Starting with the testbed and the MYNO device description, we describe the efficient transmission of an update image to constrained devices over a 6LoWPAN network.

### 5.1. Testbed

**Figure 4:**
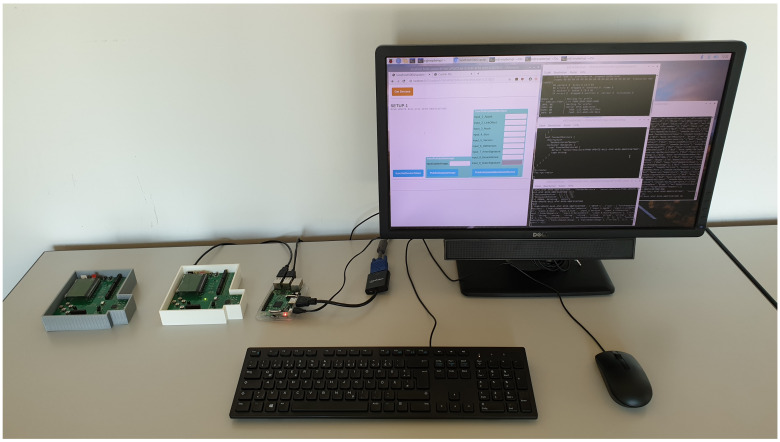
Testbed with a Raspberry Pi 3B and a CC2538dk Development Kit consisting of two CC2538EM microcontrollers plugged into the SmartRF06 Evaluation Boards and used as a 6LowPAN Border Router and an IoT device (wireless and battery-powered) on the left.

We implemented the proposed MYNO update approach in a testbed for the microcontroller board CC2538dk [39] from Texas Instruments with Contiki-NG v4.5 [40] to show the feasibility of our approach. The CC2538dk is a constrained device with an ARM Cortex-M3 processor, 32 kB RAM, 512 kB flash memory and an IEEE 802.15.4 compliant system-on-chip. Software support for 6LoWPAN is provided by Contiki-NG. One CC2538dk board is used as 6LoWPAN router and the other boards are used as IoT devices with sensors and actuators. Our testbed is shown in Figure 4. The edge node, the Raspberry Pi 3B with Raspberry Pi OS, is running an MQTT Broker (Mosquitto v1.6.10), the NETCONF-MQTT bridge and the Update Server as well as the *tunslip6* tool for tunneling the IP-Traffic over the serial port for the 6LoWPAN router.

We used the open-source free library *crypto-algorithms* [41] for computation of SHA-256 hash values. For signatures, the Elliptic Curve Digital Signature Algorithm (ECDSA) on the curve *secp256r1* is used. On the CC2538dk board, we used the library *micro-ecc* [42] for the validation of inner and outer signature. This is a small and fast ECDH and ECDSA implementation for 8-bit, 32-bit, and 64-bit processors.

### 5.2. Device Description

We extended the MYNO semantic device descriptions based on the oneM2M Ontology [25]. There are three RPC calls required which must be translated to MQTT publish messages: getDeviceToken, sendExtendedManifest, and sendFirmwareImage.

The extension of the device description for the first call, the getDeviceToken is shown as a snippet in JSON-LD format in Listing A.1 in the Appendix A. This example shows that a *Device* named myDevice has a *Service* servGetDeviceToken with an *Operation* opGetDeviceToken with MQTT properties mqttMethod and mqttTopic. Additionally, myDevice has a *Controlling Functionality* funcGetDeviceToken with a *Command* cmdGetDeviceToken. This functionality description is intended for human readability, and also the NETCONF client uses it for RPC calls generated by YANG.

The MQTT topics for the update process were defined in the device description (see Table 3). The devices expect this topic pattern and their device UUID at the end of the topics. In this way every device can be uniquely addressed via MQTT topic. The topics are used by the NETCONF-MQTT bridge for translation between RPC calls and MQTT publish and subscribe messages.

We evaluated the overhead introduced by the proposed semantic device descriptions. The size of the device description increased from 10.88 kB to 27.49 kB, since we added three controlling functions for MUP and the descriptions of the manifest parameters and error definitions as well as new MQTT topics. This device description was already reduced in size by using the compacted JSON-LD format [43]. The expanded document format has a size of 41.27 kB. The size of the device descriptions could be further reduced by RDF/HDT compression for semantic datasets as shown in [44]. However, the device description is transmitted only once, during the bootstrap process. Constrained devices send the device description in pieces to the bridge using QoS 1. The size of these pieces depends on the MQTT buffer implementation on a device. The last piece of the device description has a tag *END* so that the bridge can process the device description. Opposite to the device description which is published by the device, the firmware image is sent from the Update Server to the device.

### 5.3. Transmitting the Firmware Image

In our test setup, the firmware image had a size of 87.8 kB. Hence, the transfer of the image to the constrained device was a challenging task. Table 4 shows the communication stack of our testbed. Due to the limited resources, the communication stack and the implemented protocols try to be lightweight. Contiki-NG relies on uIP [45], the IPv6 compliant TCP/IP stack, designed to be used with tiny 8 and 16 bit microcontrollers [46].

### 5.4. Transfer via NETCONF–MQTT Bridge versus MQTT Publish

If the update image is transmitted via the NETCONF-MQTT bridge, XML RPCs will be used which results in ASCII-encoded hex strings. For example, a hexadecimal “A” (= 1010${}_{2}$) is transmitted as its ASCII code 65 (= 1000001${}_{2}$). This is highly inefficient since it doubles the image size.

Alternatively, the update image could be sent directly to the MQTT broker instead of passing it through the NETCONF-MQTT bridge. In that case, it would not have to be encoded in ASCII format and would remain at its original size. Only one RPC is needed at the beginning that instructs the device to start the verification process, as well as one RPC reply where the device indicates the result of the verification (see Listing 1).

Listing 1: Example NETCONF RPC and reply.

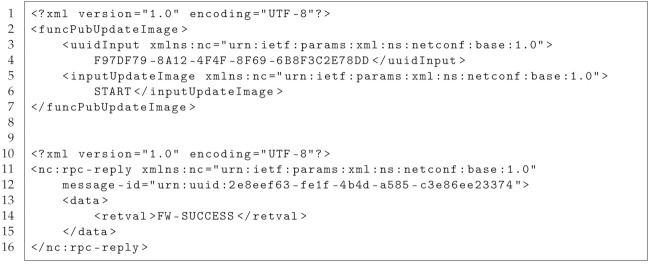



### 5.5. MQTT Slicing

Since constrained devices can receive only a limited MQTT packet size, the Maximum Packet Size property was introduced in version 5 of the MQTT protocol [4]. Thereby, a client can inform the MQTT broker about the packet size it is willing to accept. When a packet is too large, the broker must discard it without sending. However, only the MQTT broker is informed about the Maximum Packet Size value and not the application willing to publish a message intended for a such constrained device. In other words, the Update Server and the device must agree on the same packet size implementing MUP. The CC2538dk is a constrained device and the firmware image is too big for one message. Therefore, we introduced slicing on the application level for the firmware image transmission. We sliced the image like a salami into smaller packets. The slices were numbered and sent in order. Flow control was necessary to ensure the message order, as described in the next section.

### 5.6. Flow Control

An important requirement on the receiver side is that the MUP implementation on the device expects all slices to be delivered in the correct order. This approach avoids additional buffer space which would be necessary for slices that arrive out of order. Further, it simplifies the calculation of the hash values, since each received slice can be immediately piped into the hash function and processed further.

While the reliable delivery of data is typically handled by TCP, the situation is not so easy on constrained devices. For example, the uIP TCP/IP stack in Contiki-NG only allows each TCP connection to have a single TCP segment in flight at any given time.

**Figure 5:**
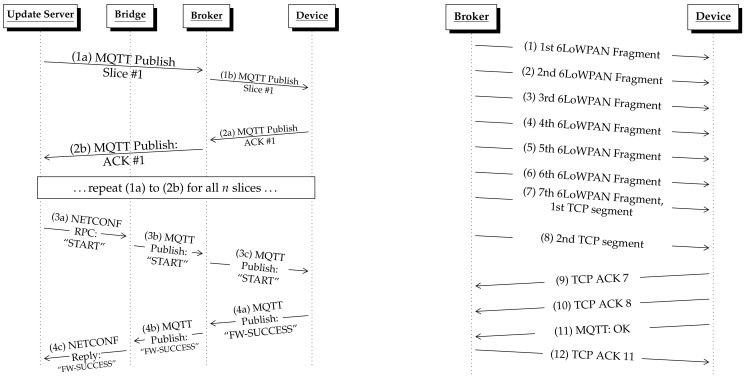
Sequence of packets sent during the transmission of a complete update file (slices 1 to *n*) (**left**). Each slice is fragmented by the 6LoWPAN Router (**right**). Example for slice size of 600 bytes.

To solve this problem, we implemented a simple *Stop-and-Wait* protocol on the application layer shown in Figure 5 left. Each slice is published to the MQTT broker, arrives at the device and is acknowledged by the MQTT client. These acknowledgments are published as MQTT messages on a response topic. When all slices have been published, a NETCONF RPC call starts the verification of the update image on the device.

The *Stop-and-Wait* protocol makes the transfer of the firmware image robust, but causes an overhead which will be analyzed in Section 6.

### 5.7. Slice Size and Fragmentation

Choosing the appropriate slice size is a challenging task. Increasing the slice size reduces the number of MQTT messages and therefore the MQTT protocol header overhead. However, it must be kept in mind that each update image slice is further fragmented by the 6LoWPAN router, since IEEE 802.15.4’s physical layer payload size is limited to 127 B. Hence, the larger the slice size, the more fragments have to be created. This also negatively impacts the performance, e.g., due to the complexity of the reassembly process and the large reassembly buffers that are required (p. 59, [47]), [48].

Figure 5 right shows this procedure for a slice size of 600 B. The slice was transmitted along with a slice number (2 B), the MQTT header with the topic name (in the example 57 B), and protocol headers (TCP/IP, 6LoWPAN). The slice was sent in two TCP segments where the first TCP segment consisted of 610 B and was divided into seven fragments, and the second segment with 54 B was small enough to fit into one IEEE 802.15.4 packet. Once the IoT device received and reassembled all fragments of a slice, it first acknowledged the receipt using a TCP ACK (message 9 and 10). It then published the MQTT response message (message 11). The broker acknowledged the receipt of the response with a TCP ACK (message 12).

The overhead due to the long MQTT topic names is a well-known problem. Hence the Topic Alias feature was introduced in MQTT v5 [4]. A Topic Alias could be set by including a two-byte integer alias with the full topic name in the first published message on any topic. All following published messages could include the alias and a zero-length topic. However, in Contiki-NG the Topic Alias feature is supported only for sending messages, but not for receiving messages.

The trade-off between slice size and fragmentation along with other performance issues is evaluated and discussed in the next section.

## 6. Performance Evaluation

Our evaluation testbed was the same as introduced in Section 5.1. We used a CC2531 USB-Dongle from Texas Instruments as a sniffer and the sniffing software *whsniff* (v1.3) [49] for traffic capture analysis. The MQTT client implementation of Contiki-NG defines several buffers which are shown in Table 5.

### 6.1. Firmware Transmission Times

We measured the firmware transmission time for three different configurations shown in Table 6. Each configuration changed one parameter compared to the previous one (marked with green color). First, we increased the slice size from 220 B to 600 B (MUP 600), next we switched the use of the response topic alias on (MUP 600 + RTA).

Each experiment was repeated three times and showed little deviation. In Table 7, the average values are shown. For each of the three configurations, the transmission time of the complete firmware is given ($t_{total}$) and the transmission time per slice. Further, the total traffic to and from the IoT device was measured.

By increasing the slice size from 220 B to 600 B, the transmission time was reduced from 146.26 s to 81.54 s. The main reason for this behavior is that the amount of traffic sent from the Update Server to the device was reduced from 199.77 kB to 150.97 kB. In the experiments, the slice size was increased by 63.33% but the *average* slice transmission duration was only increased by 37%. This was caused by the reduced fixed costs due to fewer packets sent, and hence less IP, TCP and MQTT header overhead. The incoming traffic was reduced due to the lowered header overhead, and the outgoing traffic was reduced due to the decreased number of acknowledgements that must be sent.

Further, the usage of a Topic Alias for the response topic had also a positive effect mostly on the amount of outgoing traffic, as expected. It was reduced by 31.43%. In the next subsections, we analyze the impact of the slice size on the transmission time and fragmentation overhead.

### 6.2. Impact of Slice Size

**Figure 6:**
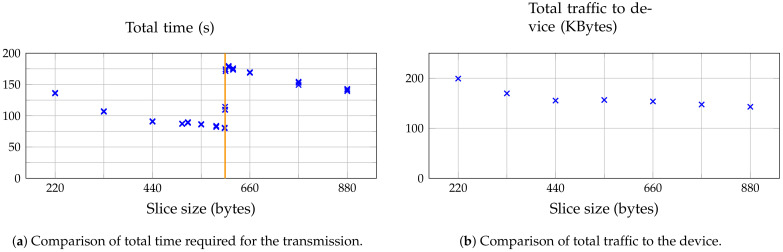
Comparison of different slice sizes. The updates were transmitted in binary encoding, using MQTT ACKs and a response topic alias.

We measured time and traffic for the firmware update with different slice sizes. Figure 6a shows the transmission time of the firmware image for different slice sizes. We did measurements for varying slice sizes between 220 B and 880 B. Larger slices caused the IoT device to get stuck during slice transmissions, presumably due to a limited number of fragments supported by Contiki-NG.

For each slice size, the results of three update runs are shown. They deviated only very slightly from each other. The transmission time lies between 79.86 s and 180 s. Larger slice sizes led to a lower total transmission time up to a threshold size of 603 B (marked with an orange line). The average transmission time measured at a slice size of 603 B was 80.45 s. The average transmission times measured at the slice sizes of 604 B and 605 B are 111.17 s and 173.22 s, respectively. An inspection of the traffic traces shows that this is due to a delay that appeared when the MQTT message lengths are larger than 607 B. The MQTT message length consists of the slice size plus the bytes used for the slice number (between 2 B and 4 B, depending on the number of digits in the slice number).

Figure 6b shows that the amount of total traffic sent to the device is much higher than the 87.8 kB firmware image. For larger slice sizes the amount of total traffic decreased steadily, but only slightly for slice sizes bigger than 440 B. For example, for a slice size of 220 B the firmware image is transmitted in 399 slices which results in 399 MQTT publish messages. For a slice size of 880 B, this number is lowered to only 99 publish messages which drastically reduced the overhead caused by the long MQTT topic names. On the other hand, the overhead due to fragmentation increased which will be discussed in detail in the next section.

### 6.3. Fragmentation Overhead

Since IEEE 802.15.4 allows only a physical layer payload size of 127 B, the image slice was fragmented by the 6LoWPAN router. To illustrate the overhead due to fragmentation, we analyzed the traffic going from the MQTT Broker to the device. The capture files were analyzed using a custom Python program based on the packet parser PyShark (version used: v0.4.2.11). PyShark [50] is a Python wrapper for tshark, a command line tool for network analysis that comes bundled with Wireshark.

**Figure 7:**
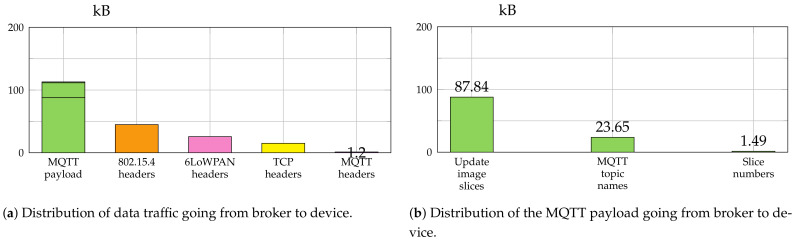
Analysis of the network traffic captured during the transmission of an update image file with a size of 87.8 kB using a slice size of 220 bytes.

**Figure 8:**
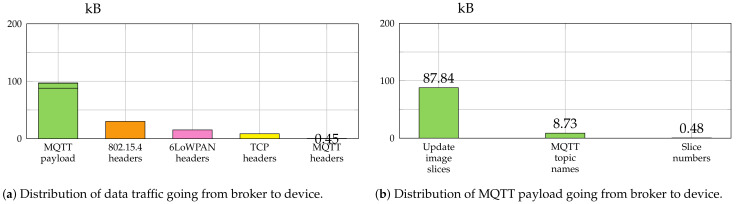
Analysis of the network traffic captured during the transmission of an update image file with a size of 87.8 kB using a slice size of 600 bytes.

Figure 7 and Figure 8 give a summary of the amount of data which was sent between broker and device for the transfer of the complete firmware image of 87.8 kB with a slice size of 220 B and 600 B, respectively. In case of slice size 220 B the border router fragmented the message into four fragments of size 120, 96, 96, and 40 B. The 600 B slice size resulted in seven fragments (120, 5 × 96 and 40 B).

The smaller slice size resulted in a total amount of traffic from broker to device of 199.74 kB, while the traffic was reduced to 151.39 kB for the bigger one. Figure 7a and Figure 8a show a detailed breakdown of the traffic going from broker to device for the protocols IEEE 802.15.4, 6LoWPAN, TCP and MQTT.

The MQTT payload is analyzed further in Figure 7b and Figure 8b. The MQTT topic names caused considerable additional traffic overhead. The bigger slice size reduced this overhead from 23.65 kB to 8.73 kB. Since fewer MQTT publish messages were necessary, about 15 kB were saved just for the MQTT topic names.

Smaller slice sizes than 220 B were not tested. It can be expected that they would only result in worse traffic and time values due to increased header overhead and time spent waiting for ACKs. We could only expect an improvement if the slice size was decreased so much that the slices would not need to be fragmented further by the 6LoWPAN router, since fragmentation caused a performance penalty (p. 59, [47]), [48]. However, this was not possible at the time of writing, since the topic name alone already caused at least two fragments to be created and topic aliases could not be implemented for the update slice topic because of the lacking Contiki-NG support.

### 6.4. Acknowledgment Traffic

**Figure 9:**
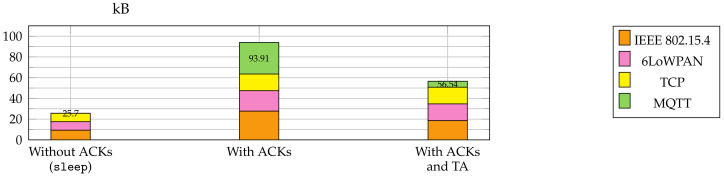
Comparison of Acknowledge Traffic from Device to MQTT Broker for the Slice Size of 220 Bytes (Total of 399 Slices) with and without Topic Alias (TA) in Response.

The *Stop-and-Wait* protocol made the implementation robust, but it was also a performance bottleneck. Figure 9 shows the acknowledgement traffic from the device to the broker for a slice size of 220 B where 399 slices had to be acknowledged. This resulted in 93.91 kB total acknowledgement traffic. Since Contiki-NG supports the Topic Alias feature for outgoing messages, we switched it on. This optimization reduced the traffic to 56.54 kB. Additionally, we increased the value for the Contiki-NG parameter MAX_TCP_SEGMENT_SIZE to 128 for outgoing messages on the device. The acknowledgement was then sent in only one TCP segment instead of two, lowering the segmentation and packet header overhead.

Since we wanted to evaluate the overhead introduced by the *Stop-and-Wait* protocol, we implemented a MUP version where a sleep was used between the slice publish messages instead of acknowledgements. The sleep time was determined experimentally to give the device enough time to process a slice completely and be ready for the next one. For slice size 220, a sleep time of 0.3 s was appropriate. This allowed sending the firmware update without any acknowledgements on MQTT level and there remained only the acknowledgements traffic on the TCP level. This reduced the amount of traffic from the device to the broker to 25.7 kB.

## 7. Discussion of MQTT Implementation Issues

There are possibilities for optimization regarding constrained devices in the MQTT protocol which will be discussed in this section.

### 7.1. Slice Size

The experiments have confirmed that a bigger slice size reduces the protocol header overhead, since fewer MQTT publish messages are sent. However, there is an upper limit for the slice size, because the communication stack is optimized for the constrained device and uses static communication buffers. While in our test environment we achieved good performance results with a slice size of 600 B, this value obviously depends on the given hardware and software, and has to be re-evaluated for other settings.

A Maximum Packet Size parameter was introduced in MQTT v5. The device may set this parameter, but the MQTT broker will not inform the publisher client. Hence, the MQTT clients (publisher and subscriber) have to agree on the same packet size in advance. Instead, our prototype implementation transfers the firmware via slices which is comparable with the block-wise transfer [51] already specified in the CoAP protocol for transferring multiple blocks of information in so-called multiple request-response pairs. For the better support of constrained devices, we propose to add a similar feature to the next MQTT version. Instead of the publisher client, the MQTT broker should be responsible for slicing to the maximum packet size specified by the device.

### 7.2. MQTT Quality of Servce

While the *Stop-and-Wait* protocol is a robust solution, it implicates overhead as shown in the detailed traffic analysis. Alternatively, Quality of Service (QoS) 1 or 2 in the MQTT protocol could be used for delivery assurance ("at least once” or "exactly once”).

Additionally, in MQTT v5 a new property Receive Maximum is defined to control the number of unacknowledged PUBLISH packets the clients receive. In combination, this would delegate the burden of the *Stop-and-Wait* protocol down to the MQTT layer and reduce the amount of traffic. Unfortunately, the current Contiki-NG v4.5 supports QoS 1 and 2 only for *outgoing* messages [30]. For incoming messages this is still an open issue in Contiki-NG which reflects that over the air firmware updates in the IoT is still not appropriately supported. At least, we could use QoS 1 to transmit the device description from device to broker without additional acknowledges.

### 7.3. MQTT Topic Alias

To use the Topic Alias, the clients (Update Server and IoT device) must specify that they wish to use MQTT v5 when connecting to the broker. Since MQTT v5 support is included in the newest development version of Contiki-NG, the usage of topic aliases for the messages published by the IoT device (i.e., the slice acknowledgments) has been implemented in the optimized version of MUP.

However, it was not possible to implement the usage of topic aliases for the update slice messages at the time of writing. First, the Update Server is a web application implemented in Python based on a framework called Flask. Flask offers an extension for integrating an MQTT client into a web application called Flask-MQTT [52]. This extension is a thin wrapper around the Eclipse Paho MQTT Client [53] which does not support MQTT v5 yet. It would need to be replaced by another MQTT client implementations that can be used in Python applications and already supports MQTT v5. The gmqtt implementation had the same problem [54], but recently fixed it in v0.6.7 [55].

Second, the MQTT broker implementation Mosquitto behaves in an unexpected way: It does not use topic aliases in outgoing messages to the subscribers, even when a topic alias was set by the publisher. Instead, it always performs a translation of incoming topic aliases back to the full topic name. Therefore, a topic alias set for the update slice topic never reaches the IoT device.

In the MQTT v5 specification, the Topic Alias Maximum property is defined as “the highest value that the client will accept as a Topic Alias sent by the Server” ([4] p. 37), which clearly implies that topic aliases were intended to be sent from the broker (“server”) to subscribers (“clients”). However, the specification does not clearly state that the broker MUST or SHOULD send topic aliases to subscribers when a Topic Alias Maximum is set. It only states that the broker must not send topic aliases to subscribers when the Topic Alias Maximum is not set or set to zero. Therefore, the Mosquitto broker implementation does not directly violate the specification. Still, the unexpected behavior was reported in the project’s issue tracker [56] and may be fixed in a future release. This may allow update slice topic aliases to be implemented in the future.

## 8. Conclusions and Future Work

Providing firmware updates for IoT devices is one of the central questions to deal with IoT security issues. We present MUP, a scalable and secure firmware update protocol for constrained IoT devices over MQTT. The MUP protocol does not rely on TLS.

MUP follows the two-phase approach also used in update frameworks like TUF [7] and UpKit [15]. The benefit of this approach is that the energy-intensive transfer of the firmware image is only initiated by the device when the freshness of the firmware is proven. The measurements with the prototype implementation show that the transmission of a firmware image of 87.8 kB can be done within 81.54 s, close to the results of UpKit [15]. This proves that the MUP approach is feasible within an MQTT-based IoT scenario. Further, the update protocol was easily integrated in our MYNO architecture which shows the flexibility of MYNO’s semantic approach. The ontology-driven device description and the MYNO source code including the implementation of the NETCONF-MQTT bridge, the Update Server and the device application are available as open-source [57].

While the proposed update protocol could easily be integrated with an MQTT based IoT scenario, the implementation showed some missing points in the MQTT v5 specification. While CoAP supports block-wise transmission, MQTT lacks this feature. In the MUP prototype implementation, the firmware update image must be sent in *slices* because of constraints in network bandwidth and memory on the device. Therefore, it was necessary to implement a *Stop-and-Wait* protocol on the application layer, since the MQTT broker in the testbed did not support any streaming capability to the IoT devices. We analyzed the impacts of slice size and fragmentation. Both have a considerable impact on the amount of data which have to be transferred and the firmware transmission time.

An improvement of the MUP update protocol will be the extension of the key roll-over of the public vendor key using DNSSec/DANE [37].

## Figures and Tables

**Table 1:** Manifest.

Vendor Manifest	
**Field**	**Description**
App ID	unique id for application
Link offset	memory address
Digest	hash value of the firmware
Size	size of the firmware in bytes
New Version	new firmware version
Old version	old firmware version
Inner signature	vendor signature
**Manifest Extension**	
Device UUID	unique device ID
Nonce	nonce generated by device
Outer signature	Update Server signature

**Table 2:** Device token.

Field	Description
Device UUID	unique device ID
Nonce	nonce generated by device
Version	current firmware version

**Table 3:** MQTT topics for publish/subscribe.

MQTT Topic	Message
mup/token/UUID	publish request for device token
mup/manifest/UUID	publish manifest
mup/firmware/UUID	publish firmware
mup/response/UUID	publish all responses from device

**Table 4:** Protocol stack.

MQTT
TCP
IPv6
6LoWPAN
IEEE 802.15.4

**Table 5:** Parameter settings in Contiki-NG.

Parameter	Value in Bytes	Description
MQTT_TCP_INPUT_BUFF_SIZE	512	size of the TCP input buffer
MQTT_TCP_OUTPUT_BUFF_SIZE	512	size of the TCP output buffer
MQTT_INPUT_BUFF_SIZE	512	buffer for MQTT Input Payload
MAX_TCP_SEGMENT_SIZE	128 (default 32)	customized buffer for Output TCP segments

**Table 6:** MYNO Update Protocol (MUP) configurations.

Configuration	Slice Size	Number of Slices	Response Topic Alias
**1. MUP 220**	220 B	399 slices	No
**2. MUP 600**	600 B	146 slices	No
**3. MUP 600 + RTA**	600 B	146 slices	Yes

**Table 7:** Measured performance metrics of the three evaluated configurations: total duration of the transmission of the entire update file ($t_{\mathrm{total}}$), average duration of the transmission of a single update slice ($\bar{t_{\mathrm{slice}}}$), total traffic over the wireless link to the IoT device (${\mathrm{traffic}}_{\mathrm{in}}$) and from the IoT device (${\mathrm{traffic}}_{\mathrm{out}}$).

Configuration	$t_{\mathrm{total}}$	$\bar{t_{\mathrm{slice}}}$	${\mathrm{traffic}}_{\mathrm{in}}$	${\mathrm{traffic}}_{\mathrm{out}}$
**1. MUP 220**	146.26 s	0.362 s	199.77 kB	93.91 kB
**2. MUP 600**	85.91 s	0.575 s	151.42 kB	44.03 kB
**3. MUP 600 + RTA**	81.54 s	0.545 s	150.97 kB	30.19 kB

## Data Availability

Data sharing not applicable.

## Appendix A

Listing A.1: Device description: getDeviceToken controlling functionality in the ontology.
